# Risk of breast cancer among daughters of mothers with diabetes: a population-based cohort study

**DOI:** 10.1186/bcr2481

**Published:** 2010-02-25

**Authors:** Olof Stephansson, Fredrik Granath, Anders Ekbom, Karin B Michels

**Affiliations:** 1 Clinical Epidemiology Unit, Department of Medicine, Solna, Karolinska University Hospital and Institute, T2, Stockholm, SE-171 76, Sweden; 2 Division of Obstetrics and Gynaecology, Department of Women's and Children's Health, Karolinska University Hospital and Institute, H2, Stockholm, SE-171 76, Sweden; 3 Obstetrics and Gynecology Epidemiology Center, Department of Obstetrics, Gynecology and Reproductive Biology, Brigham and Women's Hospital, Harvard Medical School, 75 Francis Street, Boston, MA 02115, USA; 4 Department of Epidemiology, Harvard School of Public Health, 677 Huntington Avenue, Boston, MA 02115, USA

## Abstract

**Introduction:**

Diabetes during pregnancy is related to enhanced fetal growth, which has been associated with increased breast cancer risk. Whether daughters of mothers with a diagnosis of diabetes have an increased risk of breast cancer is not known.

**Methods:**

We performed a retrospective cohort study of daughters of mothers with diabetes by linkage of the Swedish Multigeneration, Cause-of-Death and Patient Register between 1952 and 2005. Breast cancer cases were ascertained by linkage with the Swedish Cancer Register between 1958 and 2005. Standardized incidence ratios (SIRs) of breast cancer were calculated assuming a Poisson distribution for the observed cases.

**Results:**

We identified 291,360 daughters of mothers with a diagnosis of diabetes before or after birth between 1952 and 2005. Among the daughters, 7,956 cases of breast cancer were diagnosed between 1964 and 2005. The total time of follow-up was 12,173,821 person years. The expected number of breast cancer cases was 9,204, resulting in an SIR of 0.86 (95% CI, 0.85 to 0.88). The decrease in risk associated with maternal diabetes was stronger for premenopausal (< 55 years of age) than postmenopausal (≥ 55 years of age) breast cancer (SIR 0.83 and 0.91, respectively). Among daughters of mothers with diabetes, a history of breast cancer in the mother increased the risk of breast cancer in the daughter (SIR 1.43, 1.32 to 1.54).

**Conclusions:**

Daughters of mothers with a lifetime history of diabetes were at a decreased risk of breast cancer. The strongest negative association was found among premenopausal breast cancer.

## Introduction

In 1990, Trichopoulos suggested that breast cancer risk was influenced by the fetal environment, particularly variations in hormone concentrations [1]. Since then, numerous studies have assessed the association of birth weight as an integrative measure of prenatal nutrition and growth and development of breast cancer. A recent review by Michels and Xue [2] reports that the majority of studies show a positive association with an overall increased risk of 23% (95% confidence interval (CI) 13% to 34%) for high compared with low birth weight, and the association appears to be restricted to premenopausal breast cancer. While early hypotheses suspected intrauterine estrogen as a key player, IGF-1 and IGF-2 subsequently emerged as prime candidates. The diabetic environment is associated with increased levels of glucose and insulin, which lead to accelerated fetal growth and increased cell proliferation and subsequently a higher proportion of macrosomia (birth weight ≥ 4,500 g) and large-for-gestational-age infants [3]. Women with diabetes mellitus have a modest but statistically increased risk of postmenopausal breast cancer that has been related to altered concentrations of insulin, IGF, and/or endogenous sex hormones [4,5]. Because of the strong link between overweight and obesity, diabetes during pregnancy, and increased fetal growth [6], we hypothesized that daughters of mothers with a lifetime history of diabetes were at an increased risk for development of breast cancer.

## Materials and methods

### Study population

**Figure 1:**
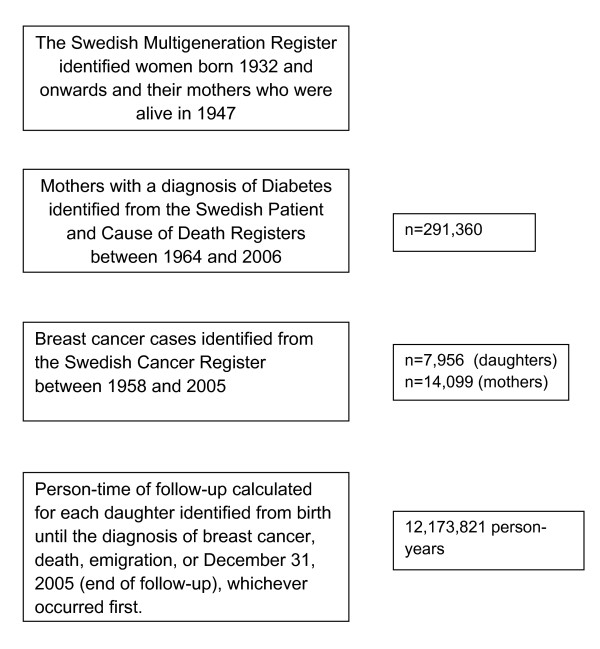
**Study design**.

The study base from which our study population was drawn is the total female Swedish population from 1932 and onwards. The unique national registration number (NRN), assigned at birth or immigration, allowed linkage to the following population-based nationwide registers: the Multigeneration Register, the Patient Register, the Cancer Register, the Register of Causes of Death, the Population Register, and the Register of Population Changes (Figure 1). The linkage provided information on all incident breast cancers in the cohort during the period 1958 to 2005, all deaths during 1952 to 2005, dates of emigration, and information about vital status for each individual through the end of the follow-up period (31 December 2005). We used the Multigeneration Register to identify women born 1932 and onwards and their mothers who were alive in 1947 (the year of the introduction of the NRN). The exposure of interest was maternal diabetes, with the index participant indicated by hospitalization for diabetes or with diabetes as a contributing cause of death. Mothers with diabetes were identified from the Patient Register and from the Cause of Death Register (defined according to the relevant International Classification of Disease (ICD) codes ICD-7: 260; ICD-8: 250; ICD-9: 250, 648W, 648A; and ICD-10: E10-E14, O24) between 1964 and 2006. Diagnosis of breast cancer for mothers and daughters between 1958 and 2005, including date of diagnosis was ascertained through the Cancer Register. Person-time of follow-up was calculated for each daughter from 1958 or birth until the diagnosis of breast cancer, death, emigration, or 31 December 2005 (end of follow-up), whichever occurred first.

The study was approved by one of the Regional Ethical Review Boards in Stockholm, Sweden. The board did not require the women to provide informed consent.

### Statistical Analysis

As the measure of association, we used standardized incidence ratios (SIRs) of breast cancer. SIRs were calculated by dividing the observed number of breast cancers with that expected, based on sex-, age-, and calendar period-specific incidence rates in the general population of Sweden. Confidence intervals of 95% (95% CI) were calculated assuming a Poisson distribution for the observed number of cases [7]. The data were analyzed with SAS software (SAS Institute Inc., Cary, NC, USA).

## Results

Through linkage among the Multigeneration Register, the Patient Register and the Cause of Death Register, we identified 291,360 daughters of mothers with a diagnosis of diabetes mellitus or gestational diabetes before or after birth from 1932 onwards. Among the daughters there were 7,956 cases of breast cancer between 1958 and 2005. The total time of follow-up was 12,173,821 person years. The expected number of breast cancer cases was 9,204, with a total SIR of 0.86 (95% CI, 0.85 to 0.88). The SIRs for breast cancer by the mother's age at diagnosis of diabetes are listed in Table 1. The lowest SIR for breast cancer was observed among maternal age at diabetes diagnosis between 50 to 59 years of age. The decrease in risk associated with maternal diabetes was stronger for premenopausal (< 55 years of age) than postmenopausal (≥ 55 years of age) breast cancer (SIR 0.83 and 0.91, respectively) (Table 2). Among the mothers there were 14,099 cases of breast cancer. Among daughters of mothers with diabetes, a history of breast cancer in the mother increased the risk of breast cancer in the daughter (SIR 1.43, 1.32 to 1.54) (Table 3). SIR for breast cancer was lowest before 1980, for all calendar periods the SIR was significantly below 1.0 (Table 4).

**Table 1:** Observed and expected breast cancers among daughters by mother's age at diagnosis of diabetes

	Number of breast cancer cases			
			
Age of mother at diagnosis of diabetes (years)	Observed	Expected	SIR	95% CI
Up to 49	911	1072	0.85	(0.80 to 0.91)
50 to 59	353	463	0.76	(0.69 to 0.85)
60 to 69	1,458	1,685	0.87	(0.82 to 0.91)
70 to 79	2,868	3,358	0.85	(0.82 to 0.89)
80 to 89	2,366	2,626	0.90	(0.86 to 0.94)
Total	7,956	9,204	0.86	(0.85 to 0.88)

CI, confidence interval; SIR, standardized incidence ratio.

**Table 2:** Observed and expected breast cancers among daughters of mothers with diabetes by age at cancer diagnosis

	Number of breast cancer cases			
			
Age of daughter at diagnosis of breast cancer	Observed	Expected	SIR	95% CI
< 55	4,430	5,332	0.83	(0.81 to 0.86)
≥ 55	3,526	3,872	0.91	(0.88 to 0.94)

CI, confidence interval; SIR, standardized incidence ratio.

**Table 3:** Observed and expected breast cancers among daughters of mothers with diabetes by family history

	Number of breast cancer cases			
			
Family history of breast cancer	Observed	Expected	SIR	95% CI
Yes	696	488	1.43	(1.32 to 1.54)
No	7,260	8,716	0.83	(0.81 to 0.85)

CI, confidence interval; SIR, standardized incidence ratio.

**Table 4:** Observed and expected breast cancers among daughters by calendar year of cancer diagnosis

	Number of breast cancer cases			
			
Year of breast cancer diagnosis	Observed	Expected	SIR	95% CI
Until1989	1,397	1,822	0.77	(0.73 to 0.81)
1990 to 1999	3,050	3,477	0.88	(0.85 to 0.91)
2000 and onwards	3,509	3,904	0.90	(0.87 to 0.93)

CI, confidence interval; SIR, standardized incidence ratio.

## Discussion

In this retrospective cohort study on close to 8,000 breast cancer cases we observed a significantly decreased risk of breast cancer among daughters of mothers with a lifetime history of diabetes. The strongest negative association was found for premenopausal breast cancer, with a 17% reduction in risk. Hence the findings from our study are in contradiction to our hypothesis of an elevated risk of breast cancer among daughters of mothers with diabetes.

Previous studies have found that increased fetal growth measured by birth weight and height is positively associated with breast cancer risk. In a meta-analysis including studies published between 1980 and 2007, Xue et al. report a small but significant 15% increased risk of breast cancer for increased birthweight and an almost 30% risk increase for high birth length [8]. The associations appeared to be stronger for premenopausal than postmenopausal breast cancer. There was no association between gestational age at birth and subsequent breast cancer risk [8].

We did not have information on gestational age and birth weight of the daughters in the study, and therefore we could not explore whether the inverse association between maternal diabetes and breast cancer risk among the daughters was influenced by these perinatal factors. Another limitation of the study is that we were not able to distinguish between type 1, type 2, and gestational diabetes among mothers. Type 2 diabetes is by far the most common diabetes type, and the underlying biologic mechanisms affecting maternal glucose metabolism are similar for all types of diabetes. We were able to identify diabetes in the mother starting in 1965. Hence, diagnosis of diabetes in the mothers could be after the birth of the daughters being followed for breast cancer. We do not know whether the mother had previously been diagnosed with diabetes, in particular, prior to her pregnancy with the daughter, or whether the hospitalization was related to newly diagnosed diabetes; it is possible that the mother developed diabetes after the index daughter was born. Since diabetes develops over a considerable time period, however, the mother is likely to have been affected by insulin resistance and hyperinsulinemia much earlier and thus also during her pregnancy with the daughter [9].

Fetal weight is strongly correlated with maternal weight in early pregnancy and weight gain during pregnancy [10]. Overweight and obese mothers are more likely to give birth to infants with a high birth weight as well as large-for-gestational-age (LGA) infants [10]. Overweight and obesity are also closely linked to gestational diabetes and type 2 diabetes later in life [6]. We postulated that diabetes would increase the risk of breast cancer among the offspring. Besides gestational and type 2 diabetes, overweight and obesity are also closely linked to development of preeclampsia [11], and the risk increases in a dose-dependent manner with increasing maternal body mass index (BMI) ([12]. Intrauterine exposure to preeclampsia has been associated with a decreased risk of breast cancer among the offspring [13,14]. Preeclampsia is linked to placental dysfunction and, as a consequence, fetal growth restriction. The placenta is the primary source of pregnancy hormones, and placental impairment may serve as an indirect marker for placental function during pregnancy. In a study by Cohn et al., markers for placental dysfunction such as low placental weight, small diameter, and presence of infarctions reduced the rate of breast cancer of the mother not explained by preeclampsia or other known breast cancer risk factors [15]. Reduced levels of estrogen by a decreased synthesis of the smaller placenta as well as increased androgen levels due to the inverse relation between placental size and androgen level may explain the reduction in breast cancer risk among women with preeclampsia [15]. Among parous women, the risk of breast cancer increases by placental weight of the pregnancies, and the association is strongest for premenopausal breast cancer [16]. Unfortunately, neither data on birth weight or placental weight nor on preeclampsia were available for the population in the current study.

Previous studies suggest a modest increase in risk of breast cancer among women with type 2 diabetes and gestational diabetes [5]. In the present study we did not have information on diabetes among the daughters and consequently we could not study its possible influence on breast cancer. We observed that diabetes in the mother had a stronger inverse association with premenopausal than postmenopausal breast cancer. Overweight and obese women have a higher incidence of postmenopausal breast cancer, whereas an inverse association has been consistently observed between BMI and premenopausal breast cancer [17]. In a large prospective Nordic study by Weiderpass et al. [18], a decreased risk of breast cancer was observed among overweight and obese women. Similar findings have been reported in a US study based on the Nurses' Health Study II [19]. The biological rationale for this decreased risk among overweight and obese women has been attributed to an increased proportion of anovulatory menstrual cycles resulting in decreased estradiol and progesterone levels [20]. However, this mechanism was not supported in the above mentioned studies [18,19]. Apart from reduced estrogen levels [21], an increased concentration of androgens and low levels of SHBG have been suggested as the biological rationale for the association between obesity and reduced breast cancer risk among premenopausal women. However, a study based on the European Prospective Investigation into Cancer and Nutrition (EPIC) reported increased risk of premenopausal breast cancer with high levels of testosterone and androstenedione [22].

Breast cancer incidence is higher in women with high socioeconomic status. In a recent UK study, the relative risk of breast cancer among the most deprived women was 16 percent lower than in the least deprived [23]. Affluent women are more likely to have their first child at a later age, have fewer children in their lifetime, and have an increased use of hormone replacement therapy (HRT), factors that have been associated with an increased risk of breast cancer. Deprived women are more likely not to attend breast cancer screening programs, which may delay cancer diagnosis [24]. However, we observed that the lowest risk of breast cancer was found prior to 1980, before the breast screening program became nationwide in Sweden. Women with low socioeconomic status have higher prevalence of overweight and obesity, which are strongly related to the development of type 2 diabetes [25]. Hence, our findings of a decreased risk of breast cancer among daughters of mothers with diabetes may also be due to confounding by socioeconomic status and for age at first birth, since we were not able to adjust for these characteristics.

By comparing breast cancer incidence in daughters of mothers with diabetes to the general population, we probably underestimate the true association because diabetes often goes undiagnosed for several years. The prevalence of diabetes in Sweden has not increased between 1986 and 1999, and would therefore not influence the results [26]. Only few cohorts worldwide allow the study of prenatal exposures and cancer risk in adult life. This is the most likely reason why the association between maternal diabetes and breast cancer has not been studied to date. The major strengths of the present study include population-based data with almost complete information on breast cancer diagnosis ascertainment and the register linkage preventing recall or reporting bias and selection bias. It is likely that we have captured the most severe cases of diabetes in the cohort; however, this may only strengthen the results, as any association will most likely be identified. Since our comparison is made with an expected number of breast cancer cases in an age-standardized population in Sweden, any misclassification due to diabetes not detected will not introduce any bias. To the best of our knowledge, no previous study has considered the possible association between maternal diabetes and subsequent breast cancer risk in the daughter. Further studies accounting for maternal as well as fetal weight, preeclampsia, and age at first birth are warranted to verify these novel findings.

## Conclusions

In conclusion we found that daughters of mothers with a lifetime history of diabetes were at a decreased risk of breast cancer. The strongest negative association was found among premenopausal breast cancer.

## Abbreviations

BMI: body-mass index; CI: confidence interval; EPIC: European Prospective Investigation into Cancer and Nutrition; HRT: hormone replacement therapy; ICD: International Classification of Disease; IGF: insulin-like growth factor; LGA: large-for-gestational-age; NRN: national registration number; SHBG: sex hormone-binding globulin; SIR: standardized incidence ratio.

## Competing interests

The authors declare that they have no competing interests.

## Authors' contributions

OS participated in the study design and interpretation of the results and drafted the manuscript. FG performed the statistical analyses. AE participated in the design of the study and interpretation of the results. KM participated in the design, interpretation of results and coordination, and helped to draft the manuscript. All authors read and approved the final manuscript.
